# Dietary Succinate Impacts the Nutritional Metabolism, Protein Succinylation and Gut Microbiota of Zebrafish

**DOI:** 10.3389/fnut.2022.894278

**Published:** 2022-05-23

**Authors:** Qianwen Ding, Chenyao Lu, Qiang Hao, Qingshuang Zhang, Yalin Yang, Rolf Erik Olsen, Einar Ringo, Chao Ran, Zhen Zhang, Zhigang Zhou

**Affiliations:** ^1^China-Norway Joint Lab on Fish Gastrointestinal Microbiota, Institute of Feed Research, Chinese Academy of Agricultural Sciences, Beijing, China; ^2^Norway-China Joint Lab on Fish Gastrointestinal Microbiota, Institute of Biology, Norwegian University of Science and Technology, Trondheim, Norway; ^3^Key Laboratory for Feed Biotechnology of the Ministry of Agriculture and Rural Affairs, Institute of Feed Research, Chinese Academy of Agricultural Sciences, Beijing, China; ^4^Norwegian College of Fishery Science, Faculty of Bioscience, Fisheries and Economics, UiT The Arctic University of Norway, Tromsø, Norway

**Keywords:** succinate, nutritional metabolism, glucose homeostasis, protein succinylation, gut microbiota

## Abstract

Succinate is widely used in the food and feed industry as an acidulant, flavoring additive, and antimicrobial agent. This study investigated the effects of dietary succinate on growth, energy budget, nutritional metabolism, protein succinylation, and gut microbiota composition of zebrafish. Zebrafish were fed a control-check (0% succinate) or four succinate-supplemented diets (0.05, 0.10, 0.15, and 0.2%) for 4 weeks. The results showed that dietary succinate at the 0.15% additive amount (S0.15) can optimally promote weight gain and feed intake. Whole body protein, fat, and energy deposition increased in the S0.15 group. Fasting plasma glucose level decreased in fish fed the S0.15 diet, along with improved glucose tolerance. Lipid synthesis in the intestine, liver, and muscle increased with S0.15 feeding. Diet with 0.15% succinate inhibited intestinal gluconeogenesis but promoted hepatic gluconeogenesis. Glycogen synthesis increased in the liver and muscle of S0.15-fed fish. Glycolysis was increased in the muscle of S0.15-fed fish. In addition, 0.15% succinate-supplemented diet inhibited protein degradation in the intestine, liver, and muscle. Interestingly, different protein succinylation patterns in the intestine and liver were observed in fish fed the S0.15 diet. Intestinal proteins with increased succinylation levels were enriched in the tricarboxylic acid cycle while proteins with decreased succinylation levels were enriched in pathways related to fatty acid and amino acid degradation. Hepatic proteins with increased succinylation levels were enriched in oxidative phosphorylation while proteins with decreased succinylation levels were enriched in the processes of protein processing and transport in the endoplasmic reticulum. Finally, fish fed the S0.15 diet had a higher abundance of Proteobacteria but a lower abundance of Fusobacteria and *Cetobacterium*. In conclusion, dietary succinate could promote growth and feed intake, promote lipid anabolism, improve glucose homeostasis, and spare protein. The effects of succinate on nutritional metabolism are associated with alterations in the levels of metabolic intermediates, transcriptional regulation, and protein succinylation levels. However, hepatic fat accumulation and gut microbiota dysbiosis induced by dietary succinate suggest potential risks of succinate application as a feed additive for fish. This study would be beneficial in understanding the application of succinate as an aquatic feed additive.

## Introduction

Succinate is an important intermediate of the tricarboxylic acid (TCA) cycle and plays a crucial role in the generation of adenosine triphosphate (ATP) by donating two hydrogens to the respiratory chain in the mitochondria (1, 2). In addition, succinate generated in the TCA cycle can be released into the cytosol by solute carrier family 25 member 10 (SLC25A10) and porins, further acting as a stabilizer of hypoxia-inducible factor-1α (HIF-1α; 3). The activation of HIF-1α enhances glycolysis and sustains the production of pro-inflammation cytokine interleukin-1 β (IL-1β; 4). The SLC13 family transports succinate across cell membranes (3). Extracellular succinate serves as the ligand for succinate receptor 1 (SUR1; 3), resulting in protein kinase C/mitogen-activated protein kinase cascade activation, phosphorylation of extracellular signal-regulated kinase, and calcium mobilization (5). Thus, not only does it serve as an energy substrate, but also succinate locates at the crossroads of signaling pathways.

Since the first purification from amber distillation by Georgius Agricola in 1,546, succinate has been produced *via* microbial fermentation for application in agricultural, food, and pharmaceutical industries (6). Especially in food and feed industries, succinate has been widely used as an acidulant, flavoring additive, and antimicrobial agent (7, 8). Dietary succinate can reduce body weight and increase the whole body energy expenditure by upregulating brown adipose tissue adipogenesis and thermogenesis in mice fed a high-fat diet (9). Also, it reduces the hepatic output of glucose but increases intestinal gluconeogenesis in mice fed high-fat and high-sugar diets (10). In addition, succinate feeding alleviates hyperglycemia in ob/ob mice and reduces hepatic steatosis (11, 12). Surplus succinate appears to be channeled into lipid storage through the generation of acetyl-CoA (13). These evidences suggest exogenous succinate can modulate glucose and lipid metabolism.

Due to the growth-promoting and antibacterial effects, organic acids, such as acetate and butyrate, have been used as antibiotic growth promoters in the culture of aquatic animals (14–16). As early as 1988, succinate in an additive amount of 12% was reported to improve protein efficiency by serving as a substrate for energy metabolism in rainbow trout (*Oncorhynchus mykiss*), albeit compromising growth and feed intake (17). Recently, an *in vitro* study has suggested that succinate could promote macrophage phagocytosis and innate immune response in tilapia (*Oreochromis niloticus*; 18). In addition, the growth- and immunity-enhancing effects of succinate (0.5% of the diet) in Pacific white shrimp (*Litopenaeus vannamei*) suggest that succinate is a potential health-promoting feed additive for shrimp (19). Increased survival was observed in juvenile black tiger shrimp (*Penaeus monodon*) fed a succinate-supplemented diet, which is related to the enhanced disease-resistant capability (20). Specially, dietary succinate increases the abundance of Proteobacteria and reduces the abundance of Bacteroidetes in Pacific white shrimp (21). However, the dietary intervention of succinate on nutrition metabolism and gut microbiota of fish remains unknown.

Zebrafish (*Danio rerio*) have been increasingly used in the evaluation of aquatic feeds due to their easy maintenance, evolutionary similarities to important cultured fish, and similar physiological changes in response to alterations in dietary ingredients (22, 23). In this study, zebrafish were used to evaluate the effects of dietary succinate on growth, energy budget, nutritional metabolism, protein succinylation, and gut microbiota composition.

## Materials and Methods

### Fish Husbandry

Zebrafish of the Tübingen strain were maintained in the zebrafish facility of the Institute of Feed Research, Chinese Academy of Agricultural Sciences (Beijing, China). The size of each tank was 25.5 cm × 18.5 cm × 18.0 cm. During the 4-week feeding period, the water in the rearing system was kept running, the rearing temperature was 25–28°C, the dissolved oxygen was >6.0 mg/L, the pH was 7.0–7.2, the nitrogen content was <0.50 mg/L, and the nitrogen content (as NO_2_) was <0.02 mg/L. Zebrafish were maintained at a 14:10 L:D cycle.

### Diets and Feeding Trial

The feed formulation is presented in Supplementary Table 1. Casein, soybean oil, and wheat flour were used as dietary protein, lipid, and carbohydrate sources, respectively. The control-check diet (CK) was supplemented with soybean oil at 60 g/kg (Supplementary Table 1). Increased levels of disodium succinate (0.05, 0.1, 0.15, and 0.2%) were compensated by decreasing equal levels of zeolite powder to prepare 0.05, 0.10, 0.15, and 0.2% succinate-supplemented diets (S0.05, S0.10, S0.15, and S0.2). All dry ingredients were ground through a 60-mesh screen. Diets were prepared by manually mixing the dry ingredients with oil and water. Each diet was extruded in a manual extruder with a 2.5-mm aperture. The pellets were air-dried and stored at −20°C in plastic bags in small quantities and were ground through a 30-mesh screen prior feeding. During the feeding trial, healthy, uniformly sized 1-month-old zebrafish (0.861 ± 0.002 *g*/20 fish) were randomly divided into five groups and fed the CK, S0.05, S0.10, S0.15, or S0.2 diet (Supplementary Table 1). Zebrafish were fed to apparent satiation two times daily at 9:00 and 16:00. The feed amount for each group was recorded accordingly.

### Sample Collection and Analysis

All fish were anesthetized with tricaine methanesulfonate (MS222) before sampling. At the end of the 4-week feeding trial, the survival rate in each group was recorded (100 × final survival individuals/initial individuals); the fish in each tank were weighed to calculate weight gain [100 × (final body weight−initial body weight)/initial body weight] and feed efficiency [100 × (final body weight−initial body weight)/feed intake]. Whole fish were collected for analysis of whole body proximate composition and whole body energy gain. Intestine, liver, and muscle samples were collected for the detection of metabolic intermediates, gene expression, and protein succinylation. Intestine samples were isolated for the detection of digestive enzyme activities. Randomly selected living zebrafish from each group were used to evaluate standard metabolic energy, feeding metabolic energy, fasting plasma glucose, and glucose tolerance.

### Whole Body Proximate Composition Analysis

After the 4-week feeding trial, zebrafish were anesthetized with MS222. After removal of water from the body surface, zebrafish were weighed and frozen in liquid nitrogen. Body proximal composition was detected as previously described (24, 25). Moisture contents were determined by an oven drying method. Whole body protein contents were determined by a semiautomatic Kjeldahl system. Whole body fat contents were determined using the Soxtec method with petroleum ether as the solvent. Ash contents were determined by a high-temperature burning method.

### Determination of Whole Body Energy Gain

The energy values of fish body were measured with a calorimeter. Initial fish samples were collected at the beginning of feeding. Final fish samples were collected at the end of a 4-week feeding trial. All samples were frozen at −80°C and subject to freezing-drying. The dry weight of each sample was recorded. Energy gain (%) = (final energy value−initial energy value)/initial energy value × 100%.

### Evaluation of Metabolic Energy

Standard and feeding metabolic energy were assessed by oxygen consumption (OC) monitored in an 8-chamber (2.3 L) respirometer (15, 25). Before being transferred to the chambers, zebrafish were fasted for 24 h. An empty chamber was used as a blank control. Then, zebrafish were fed equal amount of CK or S0.15 diet for 3 days. On the 4th day, the water in the chambers was changed and initial oxygen contents in water were measured. After the transfer, the fish were fed for one more day. Final oxygen contents in water were measured after 24 h (the 5th day). OC measured during the 24-h period reflected total metabolism. Then, the zebrafish were fasted for the following 3 days (the 5th to 7th day). On the 8th day, the water in chambers was changed and initial oxygen contents in the water were measured. Then, the fish were fasted for another day. Final oxygen contents in the water were measured after 24 h (the 9th day). OC measured during the 24-h period reflected standard metabolism. Oxygen was monitored using a polarographic oxygen electrode with a temperature sensor (lower limit: 4.0 mg/L, Yellow Springs Instruments, Inc.). OC was converted to energy by using a conversion factor of 13.84 J/mg O_2_. Feeding metabolic energy (J/day) = total metabolic energy − standard metabolic energy.

### Detection of Digestive Enzyme Activities

Intestine samples were collected 4 h after the last feeding and homogenized in sterile PBS (wt:vol = 1:9). Homogenates were centrifuged at 13,201 *g* at 4°C for 10 min, and supernatants were collected for the detection of digestive enzyme activities. Enzyme activities were analyzed with the SynergyMX Multi-Functional MPP Detector (Biotek, United States) as previously described (15). Digestive enzyme activities were expressed as activity unit per gram protein (U/g protein).

### Automated Measurement of Larvae Swimming Activities

The swimming activities of zebrafish larvae fed CK or S0.15 diet (Supplementary Table 2) were measured for 1 week using an infrared (IR) tracking device (WMicroTracker; 15, 26). Zebrafish larvae were distributed into a 24-well microplate at a density of 1 larva per 2 ml medium. The swimming activities of the larvae were monitored for 72 h. Zebrafish larvae were fed two times daily (9:00, 17:00). Swimming activities were calculated by the cumulative times of activity events happened in every 30 min.

### Detection of Succinate, Pyruvate, and Acetyl-CoA

Intestine, liver and muscle samples were collected for the colorimetric or fluorescent measurement of succinate, pyruvate or acetyl-CoA (Sigma-Aldrich, United States) using 96-well plate and with the SynergyMX Multi-Functional MPP Detector (Biotek, United States). Standards for succinate or pyruvate (0, 2, 4, 6, 8, and 10 μl of 1 mM solutions) were pipetted into standard wells generating a concentration gradient of 0, 2, 4, 6, 8, and 10 nmol/well. Succinate or pyruvate assay buffer was then added to each standard well to bring the total volume to 50 μl. Tissues were rapidly homogenized in 100 μl of ice-cold succinate or pyruvate assay buffer and subjected to centrifugation at 10,000 *g* for 5 min to remove insoluble materials. Supernatants (50 μl) were mixed with 50 μl of the reaction solution and incubated for 30 min at 37°C in the dark. Absorbances were measured at 450 nm for succinate and 570 nm for pyruvate. Concentrations were calculated according to the standard curve and expressed as nanomole per milligram tissue protein (nmol/mg protein).

Standards for fluorescent detection of acetyl-CoA (0, 10, 20, 30, 40, and 50 μl of 2 μM standard solution) were placed into a black 96-well plate, generating concentrations of 0, 20, 40, 60, 80, and 100 pmol/well. Acetyl-CoA assay buffer was then added to each standard well to bring the total volume to 50 μl. Tissues were rapidly homogenized in 100 μl of ice-cold acetyl-CoA assay buffer and subjected to centrifugation at 10,000 *g* for 10 min to remove insoluble materials. The supernatants (50 μl) were mixed with 50 μl of reaction solution and incubated for 10 min at 37°C in the dark. Fluorescences were measured using an extinction/emission wavelength of 535/587 nm. The level of acetyl-CoA was expressed as nanomole per milligram tissue protein (nmol/mg protein).

### Detection of Triacylglycerol (TG)

Tissue TG was extracted by chloroform: methanol (2:1). Fresh tissues were homogenized in 1 ml of PBS and then vortexed with 5 ml of chloroform: methanol (2:1) vigorously. After standing for 10 min, the TG-containing bottom-layer was extracted and solvent was evaporated at 70°C under nitrogen. Then, 1% Triton × 100 (prepared in chloroform) was added to solubilize the TG. The mixture was then heated at 70°C and again dried under nitrogen following the addition of deionized water to disperse the TG. The TG content was determined by a quantitative enzymatic method as described in a previous study (27) and was expressed as TG weight per gram tissue (mg/g tissue).

### Detection of Plasma Glucose and Test of Glucose Tolerance

Zebrafish were fasted for 72 h. Groups of zebrafish were then used to collect blood using a standard sampling protocol (28). Briefly, blood samples were collected in sodium heparin-coated tubes from the zebrafish following cutting off the tail. Plasma was then produced by centrifugation (1,467 g/min for 10 min). The remaining zebrafish were then intraperitoneally injected with glucose [1 g/kg body weight (b.w.)] for the glucose tolerance test. Blood was collected after 1, 2, 4, 5, and 6 h. Plasma glucose was measured using the Glucose Assay kit (Nanjing Jiancheng Bioengineering Institute, China) according to the manufacturer’s instructions. Plasma glucose was expressed as mmol of glucose per liter plasma (mmol/L).

### Total RNA Extraction, Reverse Transcription, and *q*PCR

Total RNA extraction, reverse transcription, and *q*PCR were conducted as described previously (29). Total RNA was isolated using the Trizol reagent (Cwbio, Beijing, China) and then reversed transcribed to cDNA by FastKing gDNA Dispelling RT SuperMix (Tiangen, Beijing, China). The *q*PCR was performed using SYBR^®^Green Supermix according to the manufacturer’s instructions (Tiangen, Beijing, China). The results were stored, managed, and analyzed using LightCycler 480 software (Roche, Basel, Switzerland). The *q*PCR primers used are listed in Supplementary Table 3.

### Analysis of Protein Succinylation

The assay of protein succinylation was performed by Jingjie PTM BIO (Hanzhou, China). Intestine and liver samples were grinded by liquid nitrogen to cell powder and then lysed with lysis buffer (3 μM trichostation and 50 mM nicotinamide), followed by centrifugation at 12,000 *g* at 4°C for 10 min to remove debris. Then, the supernatants were collected and protein concentrations were determined with the BCA kit according to the manufacturer’s instructions (Sigma, United States). To enrich the modified peptides, tryptic peptides dissolved in NETN buffer (100 mM NaCl, 1 mM EDTA, 50 mM Tris–HCl, 0.5% NP-40, and pH 8.0) were incubated with pre-washed antibody beads (PTM402, PTM Bio) at 4°C overnight with gentle shaking. Then, the beads were washed four times with NETN buffer and two times with H_2_O. The bound peptides were eluted from the beads with 0.1% trifluoroacetic acid.

For liquid chromatography with tandem mass spectrometry (LC-MS/MS) analysis, the resulting peptides were desalted with C18 ZipTips (Millipore). The tryptic peptides were dissolved in 0.1% formic acid (solvent A), directly loaded onto a home-made reversed-phase analytical column (15 cm length, 75 μm i.d.). The gradient comprised an increase from 6 to 22% solvent B (0.1% formic acid in acetonitrile) over 43 min, 22 to 30% in 13 min, and climbing to 80% in 2 min and then holding at 80% for the last 2 min, all at a constant flow rate of 450 nl/min in an NanoElute UPLC system. The peptides were subjected to a capillary ion source for ionization and analyzed by TIMS-TOF Pro mass spectrometry. The electrospray voltage applied was 1.6 kV. The m/z scan range of the secondary mass spectrometry was 100 to 1,700. The data acquisition mode is parallel cumulative serial fragmentation mode. The resulting MS/MS data were processed with the Maxquant search engine (v.1.6.6.0). Tandem mass spectra were searched against *Danio_rerio*_7955_PR_20191112 uniprot database concatenated with the reverse decoy database. Trypsin/P was specified as the cleavage enzyme, allowing up to two missed cleavages. The mass tolerance for precursor ions was set to 20 and 20 ppm in the First search and in the Main search, and the mass tolerance for fragment ions was set to 0.02 Da. Carbamidomethyl on Cys was specified as a fixed modification, and acetylation modification and oxidation on Met were specified as variable modifications. False discovery rate (FDR) was adjusted to <1%.

### Gut Microbiota Analysis

Gut contents were collected at 4–6 h after the last feeding and were used to analyze gut microbiota composition. The 16s V3–V4 region was amplified using primer pairs 338F (5′-ACTCCTACGGGAGGCAGCAG-3′) and 806R (5′-GGACTACHVGGGTWTCTAAT-3′). 16S ribosomal RNA gene sequencing was performed by Majorbio Bio-Pharm Technology Co. Ltd. (Shanghai, China) using the Illumina MiSeq PE300 platform (Illumina, San Diego, United States). Then, the raw pair-end readings were subjected to a quality-control procedure using the UPARSE-operational taxonomic unit (OTU) algorithm (30). The qualified reads were clustered to generate OTUs at the 97% similarity level using the USEARCH sequence analysis tool (30). A representative sequence from each OTU was assigned to a taxonomic level in the Ribosomal Database Project (RDP) database using the RDP classifier (31).

### Data Analysis

Statistical analyses were conducted using GraphPad Prism 5 software (GraphPad Software, Inc., San Diego, CA, United States). The results are expressed as means ± standard errors of the means (SEMs). Comparisons between two groups were analyzed using the Student’s *t*-test, and comparisons between multiple groups were analyzed by one-way analysis of variance followed by Duncan’s test. Statistical significance was set at *p* < 0.05.

## Results

### Dietary Succinate Promotes Feed Intake and Growth

**FIGURE 1:**
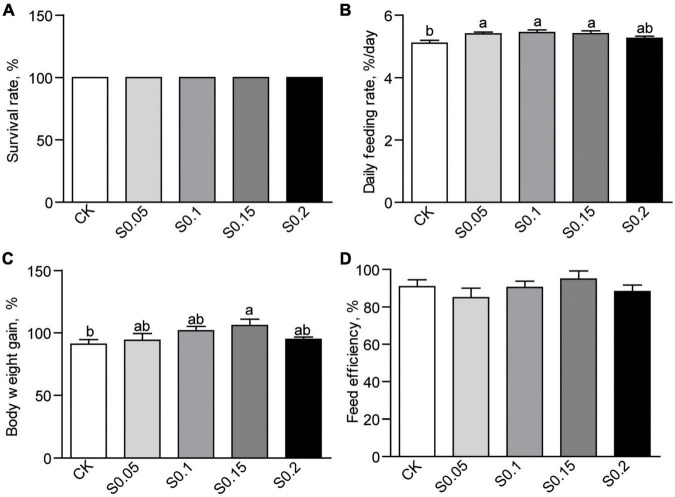
**(A)** Survival, **(B)** feed intake, **(C)** body weight gain, and **(D)** feed efficiency in zebrafish fed the CK or S0.15 diet for 4 weeks. Values are means ± standard errors of the means (SEMs; *n* = 4 biological replicates). Means without a common letter are significantly different, *p* < 0.05. CK, control-check diet; S0.05, S0.1, S0.15, and S0.2, 0.05, 0.1, 0.15, and 0.2% succinate-supplemented diets.

After the 4-week feeding trial, no mortality was observed in any of the groups (Figure 1A). Daily feeding rates were increased significantly (*p* < 0.05) with 0.05, 0.1, or 0.15% succinate supplementation, but non-significantly increased with the additive amount of 0.2%, suggesting an appetite-promoting effect of succinate (Figure 1B). Body weight tended to increase with inclusion (0.05 and 0.1%) up to 0.15%, which was significantly higher than that in fish fed the CK diet (105.7 vs. 90.9%, *p* < 0.05; Figure 1C). There were no differences in weight gain between the S0.2 and the other groups (Figure 1C). Feed efficiency was the same among all groups (Figure 1D). Thus, 0.15% succinate was used as the optimal dose in the following experiments.

### Dietary Succinate Alters Fish Body Composition and Energy Budget

**FIGURE 2:**
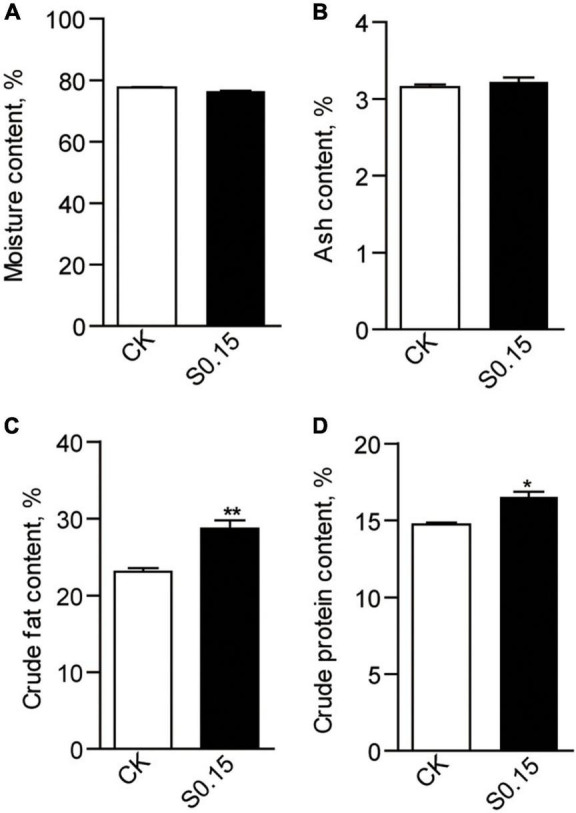
Whole body composition in zebrafish fed the CK or S0.15 diet for 4 weeks. **(A)** Whole body moisture, **(B)** whole body ash, **(C)** whole body crude fat, and **(D)** whole body crude protein. Values are means ± SEMs (*n* = 3 biological replicates). *p < 0.05, **p < 0.01. CK, control-check diet; S0.15, 0.15% succinate-supplemented diet.

The chemical composition of the whole body was analyzed after a 4-week feeding trial. Moisture and crude ash contents were similar between the CK and S0.15 group (Figures 2A,B), while crude lipid (23.1% vs. 28.7%, *p* < 0.01) and protein contents (14.74 vs. 16.52%, *p* < 0.05) were significantly increased in fish fed the S0.15 diet (Figures 2C,D).

**FIGURE 3:**
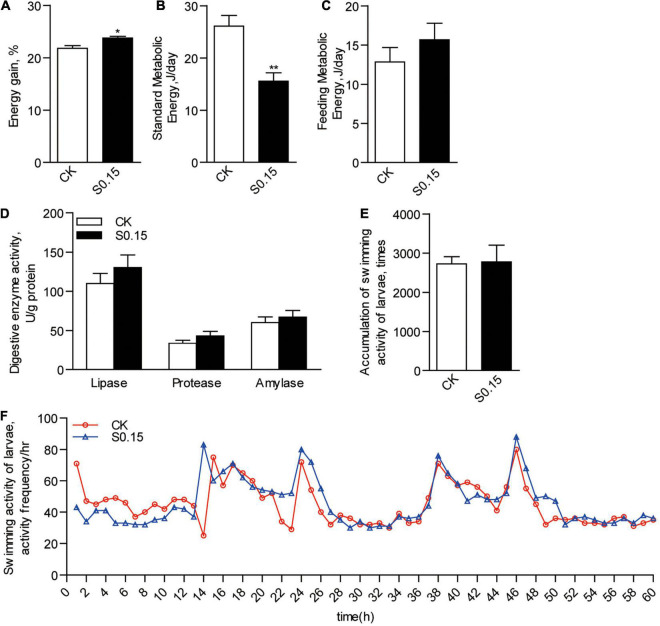
Energy budget in zebrafish fed the CK or S0.15 diet for 4 weeks. **(A)** Whole body energy gain. **(B)** Standard metabolic energy. **(C)** Feeding metabolic energy. **(D)** Digestive enzyme activities. **(E)** Accumulation of swimming activities and **(F)** representative swimming activities of zebrafish larvae fed the CK or S0.15 diet for 1 week. Values are means ± SEMs (*n* = 4–6 biological replicates). *p < 0.05, **p < 0.01. CK, control-check diet; S0.15, 0.15% succinate-supplemented diet.

Accordingly, zebrafish fed the S0.15 diet had significantly higher energy gain than those fed the CK diet (23.8 vs. 21.8%, *p* < 0.05; Figure 3A). Standard metabolic energy in the S0.15 group was 40.3% lower than that in the CK group (*p* < 0.01; Figure 3B). However, feeding metabolic energy (Figure 3C), digestive enzyme activities (lipase, protease, and amylase; Figure 3D), and swimming activities (Figures 3E,F) were similar between the two groups. These results indicate that dietary succinate could promote fat, protein, and energy deposition in the whole body of zebrafish.

### Dietary Succinate Alters the Levels of Metabolic Intermediates

**FIGURE 4:**
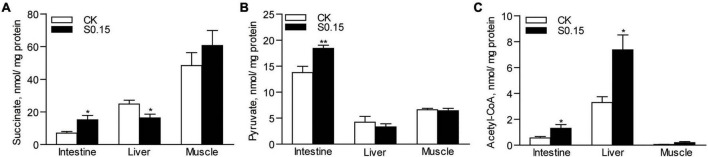
Metabolic intermediates in zebrafish fed the CK or S0.15 diet for 4 weeks. **(A)** Intestinal, hepatic, and muscular succinate levels. **(B)** Intestinal, hepatic, and muscular pyruvate contents. **(C)** Intestinal, hepatic, and muscular acetyl-CoA contents. Values are means ± SEMs (*n* = 5 biological replicates). *p < 0.05, **p < 0.01. CK, control-check diet; S0.15, 0.15% succinate-supplemented diet.

Compared with the CK group, the S0.15 group showed a significantly increased level of succinate in the intestine (2.2-fold, *p* < 0.05), but a reduced level in the liver (0.66-fold, *p* < 0.05), with no change in the muscle (Figure 4A). The level of pyruvate was significantly increased in the intestine (1.3-fold, *p* < 0.01) of the S0.15 group, with no differences in both the liver and muscle compared with the CK group (Figure 4B). The levels of acetyl-CoA were significantly elevated in the intestine (2.3-fold, *p* < 0.05) and liver (2.2-fold, *p* < 0.05) of the S0.15 group, with no changes in the muscle (Figure 4C). These results suggest that dietary succinate regulates the levels of metabolic intermediates in the intestine and liver.

### Effects of Dietary Succinate on Fat Accumulation and Glucose Homeostasis

**FIGURE 5:**
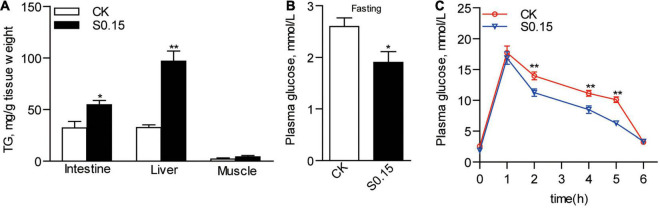
Fat accumulation and glucose homeostasis in zebrafish fed the CK or S0.15 diet for 4 weeks. **(A)** Intestinal, hepatic, and muscular TG levels. **(B)** Fasting plasma glucose levels. **(C)** Glucose tolerance test. Values are means ± SEMs (*n* = 4–21 biological replicates). *p < 0.05, **p < 0.01. CK, control-check diet; S0.15, 0.15% succinate-supplemented diet.

Zebrafish fed the S0.15 diet had 54.5 and 96.8 mg of TG per gram tissue in the intestine and liver, respectively, (Figure 5A). This was significantly higher than that in the CK group, which had only 32.1 and 32.4 mg/g TG in the same tissues, respectively (*p* < 0.05; Figure 5A). Feeding the S0.15 diet apparently increased hepatic fat contents in the intestine and liver, as shown by H&E and oil red O staining (Supplementary Figures 1A–D). However, no significant changes in muscle TG content were observed in the S0.15 group (Figure 5A and Supplementary Figure 1E). The basal plasma glucose level in zebrafish fed the S0.15 diet was significantly lower than those fed the CK diet (1.9 vs. 2.6 mM, *p* < 0.05; Figure 5B). Moreover, feeding the S0.15 diet significantly improved glucose tolerance, as shown by the significantly higher rates of glucose clearance when monitored at 2–6 h after intraperitoneal injection (*p* < 0.01; Figure 5C). These results demonstrate that dietary succinate could promote hepatic and intestinal fat accumulation while improve glucose homeostasis.

### Dietary Succinate Regulates the Expression of Lipid Metabolism-Related Genes

**FIGURE 6:**
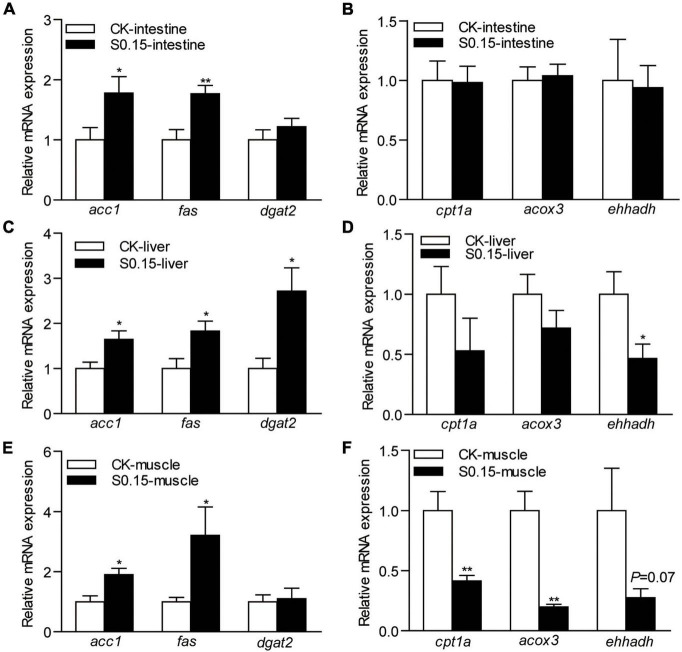
The expression of lipid metabolism-related genes in zebrafish fed the CK or S0.15 diet for 4 weeks. **(A)** Lipid synthesis- and **(B)** lipid-degradation-related genes in the intestine. **(C)** Lipid synthesis- and **(D)** lipid-degradation-related genes in the liver. **(E)** Lipid synthesis- and **(F)** lipid-degradation-related genes in the muscle. Values are means ± SEMs (*n* = 6 biological replicates). *p < 0.05, **p < 0.01. CK, control-check diet; S0.15, 0.15% succinate-supplemented diet.

The expression levels of intestinal lipid synthesis-related genes *acetyl-CoA carboxylase 1* (*acc1*; 1.8-fold) and *fatty acid synthase* (*fas*; 1.8-fold) were significantly elevated in zebrafish fed the S0.15 diet (*p* < 0.05; Figure 6A). Of the lipid-degradation-related genes, *carnitine palmitoyltransferase 1a* (*cpt1a*), *acyl-CoA oxidase 3* (*acox3*), and *enoyl-CoA hydratase and 3-hydroxyacyl CoA dehydrogenase* (*ehhadh*) were not affected by the S0.15 diet compared with the CK diet (*p* > 0.05; Figure 6B).

In the liver, the expression levels of lipid synthesis-related genes *acc1* (1.6-fold), *fas* (1.8-fold), and *diacylglycerol acyltransferase 2* (*dgat2*; 2.7-fold) were significantly increased by S0.15 feeding (*p* < 0.05; Figure 6C). The marker for lipid-degradation *ehhadh* (0.47-fold, *p* < 0.05) was significantly reduced in zebrafish fed the S0.15 diet, while the other markers tested, *cpt1a* and *acox3* were not regulated (Figure 6D).

In the muscle, lipid synthesis-related genes *acc1* (1.9-fold, *p* < 0.05) and *fas* (3.2-fold, *p* < 0.05) were significantly upregulated in fish fed the S0.15 diet while *dgat2* was not affected (Figure 6E). The expression levels of lipid-degradation-related genes *cpt1a* (0.41-fold, *p* < 0.01) and *acox3* (0.2-fold, *p* < 0.01) were significantly downregulated in zebrafish receiving the S0.15 diet, while *ehhadh* (0.27-fold, *p* = 0.07) was not affected although it had a clear tendency of downregulation in the S0.15 group (Figure 6F). These results indicate that dietary succinate might promote lipid anabolism in the intestine, liver, and muscle.

### Dietary Succinate Regulates the Expression of Glucose Metabolism-Related Genes

**FIGURE 7:**
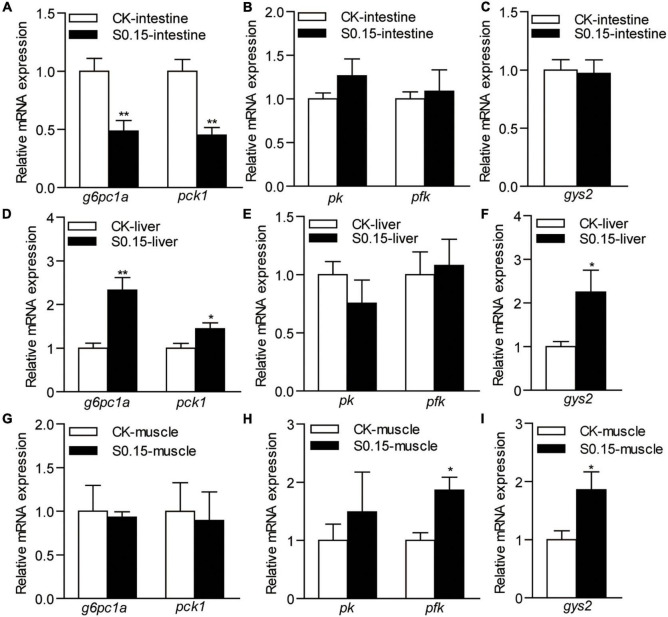
The expression of glucose metabolism-related genes in zebrafish fed the CK or S0.15 diet for 4 weeks. **(A)** Gluconeogenesis-, **(B)** glycolysis-, and **(C)** glycogen synthesis-related genes in the intestine. **(D)** Gluconeogenesis-, **(E)** glycolysis-, and **(F)** glycogen synthesis-related genes in the liver. **(G)** Gluconeogenesis-, **(H)** glycolysis-, and **(I)** glycogen synthesis-related genes in the muscle. Values are means ± SEMs (*n* = 6 biological replicates). *p < 0.05, **p < 0.01. CK, control-check diet; S0.15, 0.15% succinate-supplemented diet.

The expression levels of intestinal genes involved in gluconeogenesis *glucose-6-phosphatase catalytic subunit 1a* (*g6pc1a*; 0.49-fold) and *phosphoenolpyruvate carboxykinase 1* (*pck1*; 0.45-fold) were significantly downregulated in zebrafish fed the S0.15 diet (*p* < 0.01; Figure 7A), whereas the expression levels of the genes in the glycolysis and glycogen synthesis pathways, *phosphorylase kinase* (*pk*), *phosphofructokinase* (*pfk*), and *glycogen synthase 2* (*gys2*), were not affected by the S0.15 diet (Figures 7B,C).

In the liver, the expression levels of the hepatic gluconeogenesis-related genes *g6pc1a* (2.33-fold) and *pck1* (1.44-fold) were significantly upregulated when fed the S0.15 diet (*p* < 0.05; Figure 7D), while the expression levels of glycolysis-related genes showed no change (Figure 7E). Glycogen synthesis-related gene *gys2* was significantly upregulated in the S0.15 group (2.25-fold, *p* < 0.05; Figure 7F) compared with the CK group.

In the muscle, there were no effects of the S0.15 diet on the expression of gluconeogenesis-related genes (Figure 7G), whereas glycolysis-related genes *pfk* (1.87-fold, *p* < 0.05) increased with S0.15 feeding and *pk* had a non-significant increase as did (Figure 7H). The expression of glycogen synthesis-related gene *gys2* was significantly upregulated in the S0.15 group (1.86-fold, *p* < 0.05; Figure 7I) compared with the CK group. These results indicate that dietary succinate could inhibit intestinal gluconeogenesis but promote hepatic gluconeogenesis. In addition, glucose utilization in the liver and muscle might be increased by succinate feeding.

### Dietary Succinate Regulates the Expression of Protein Metabolism-Related Genes

**FIGURE 8:**
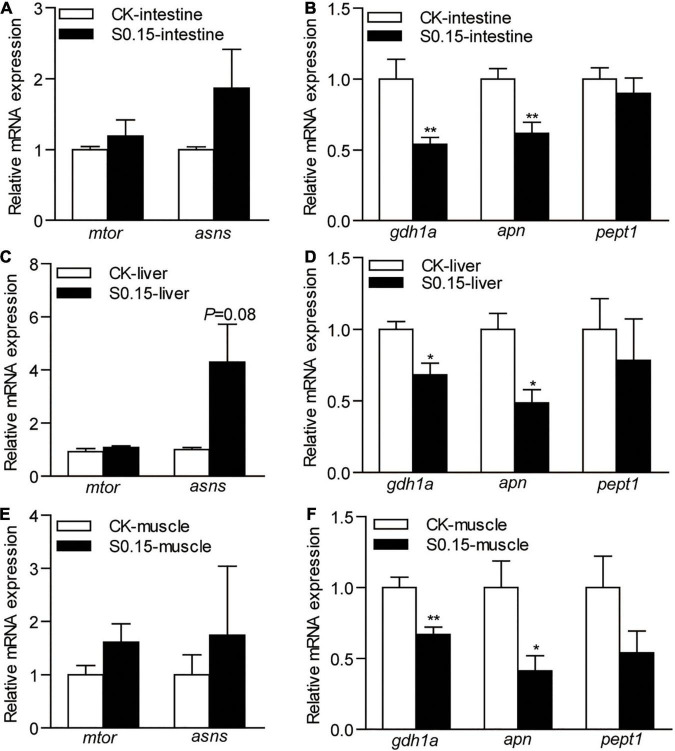
The expression of protein metabolism-related genes in zebrafish fed the CK or S0.15 diet for 4 weeks. **(A)** Protein synthesis- and **(B)** protein degradation-related genes in the intestine. **(C)** Protein synthesis- and **(D)** protein degradation-related genes in the liver. **(E)** Protein synthesis- and **(F)** protein degradation-related genes in the muscle. Values are means ± SEMs (*n* = 6 biological replicates). *p < 0.05, **p < 0.01. CK, control-check diet; S0.15, 0.15% succinate-supplemented diet.

The expression of intestinal genes related to protein synthesis, *mammalian target of rapamycin* (*mtor*), and *asparagine synthetase* (*asns*), were not affected by the S0.15 diet (Figure 8A), while protein degradation-related genes *glutamate dehydrogenase 1a* (*gdh1a*; 0.54-fold), *aminopeptidase N* (*apn*; 0.62-fold) were significantly downregulated by S0.15 feeding (*p* < 0.01; Figure 8B) compared with CK feeding.

Similarly, the expression of the same genes related to hepatic protein synthesis was not significantly affected by the S0.15 diet (Figure 8C), while S0.15 feeding significantly downregulated protein degradation-related genes *gdh1a* (0.68-fold) and *apn* (0.48-fold) compared to CK feeding (*p* < 0.05; Figure 8D).

The expression levels of the same genes related to muscular protein synthesis were not significantly affected by diets (Figure 8E). As in the intestine and liver, the S0.15 diet appeared to downregulate the gene markers for protein degradation *gdh1a* (0.67-fold, *p* < 0.01) and *apn* (0.41-fold, *p* < 0.05), as well as a non-significant reduction of *pept1* expression in the muscle (Figure 8F). These results suggest that dietary succinate could inhibit protein catabolism of the intestine, liver, and muscle, suggesting protein-sparing effects.

### Dietary Succinate Alters the Protein Succinylation Pattern in the Intestine

The distribution of intestinal protein succinylation in zebrafish fed the CK or the S0.15 diet was confirmed by western blotting using an anti-succinyllysine antibody (Supplementary Figure 2A). According to the western blotting results, the molecular weight of intestinal succinylated proteins was 25–100 kD (Supplementary Figure 2A). Upon S0.15 feeding, intestinal proteins with a molecular weight of 25–35 kD had increased succinylation levels, while proteins with a molecular weight of 35–70 kD had decreased succinylation levels compared with CK feeding (Supplementary Figure 2A). A total of 6,949 succinylated peptides were identified from the 28,608 MS/MS spectra with a FDR of <1%, and 1,644 succinylated lysine sites were identified in 588 succinylated proteins. The summary of all differentially modified proteins and sites in the intestine is presented in Supplementary Figure 2B. Among the intestinal proteins, succinylation levels of 41 sites of 29 proteins were upregulated, while succinylation levels of 29 sites of 19 proteins were downregulated in the S0.15 group (Supplementary Figure 2B) compared to the CK group.

### Dietary Succinate Alters the Protein Succinylation Pattern in the Liver

Western blotting results showed that the molecular weight of hepatic succinylated proteins was 25–130 kD (Supplementary Figure 2C). Upon S0.15 feeding, hepatic proteins with a molecular weight of 25–35 kD had increased succinylation levels, while proteins with a molecular weight of 70–130 kD had decreased succinylation levels compared with CK feeding (Supplementary Figure 2C). A total of 8,751 succinylated peptides were identified from the 36,755 MS/MS spectra with a FDR of <1%, and 1,481 succinylated lysine sites were identified in 483 succinylated proteins. The summary of all differentially modified proteins and sites in the liver is presented in Supplementary Figure 2D. Compared with the CK group, only nine sites of eight proteins in the S0.15 group showed upregulated succinylation levels, while 59 sites of 49 proteins showed downregulated succinylation levels (Supplementary Figure 2D).

### Protein Succinylation Is Involved in Intestinal Metabolic Pathways

**FIGURE 9:**
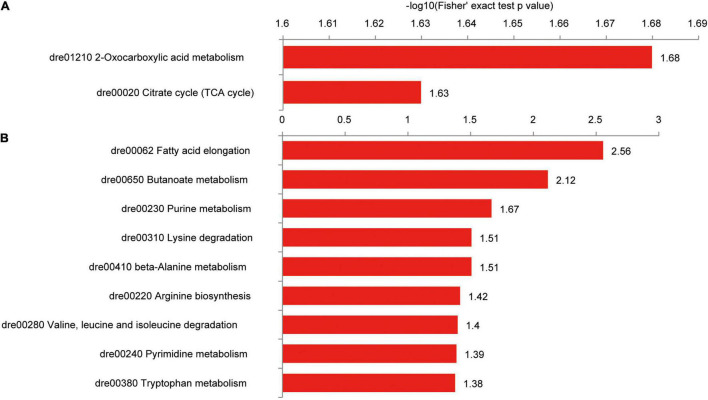
KEGG pathways of differentially succinylated proteins in the intestine of zebrafish fed the CK or S0.15 diet for 4 weeks. **(A)** Pathways involved in proteins with increased succinylation. **(B)** Pathways involved in proteins with decreased succinylation.

In the Kyoto Encyclopedia of Genes and Genomes (KEGG) pathway analysis, intestinal proteins with upregulated succinylation levels were enriched in oxocarboxylic acid metabolism and TCA cycle (Figure 9A), including isocitrate dehydrogenase 3b (Idh3b), citrate synthase (Cs), glutamic-oxaloacetic transaminase 2b (Got2b), Pck2, and malate dehydrogenase (Mdh2). Proteins with downregulated succinylation levels were part of pathways for fatty acid and amino acid degradation, including hydroxyacyl-CoA dehydrogenase trifunctional multienzyme complex subunit alpha a (Hadhaa), Hadhab, and enoyl-CoA hydratase, short chain 1 (Echs1; Figure 9B).

Among the intestinal proteins with increased succinylation levels, Cs was highly interactive with other proteins, including Mdh2, acyl-CoA dehydrogenase medium chain, Idh3b, Got2b, Pck2, heat shock protein family D member 1 (Hspd1), and ATP synthase alpha subunit (Atp5a1; Supplementary Figure 3A). Similarly, a network of interaction was formed among proteins with reduced succinylation levels, including superoxide dismutase 2, hydroxysteroid 17-beta dehydrogenase 10 (Hsd17b10), Hadhaa, Hadhab, and Echs1 (Supplementary Figure 3B). These results suggest that dietary succinate could negatively or positively influence intestinal protein succinylation levels.

### Proteion Succinylation Is Involved in Hepatic Metabolic Pathways

**FIGURE 10:**
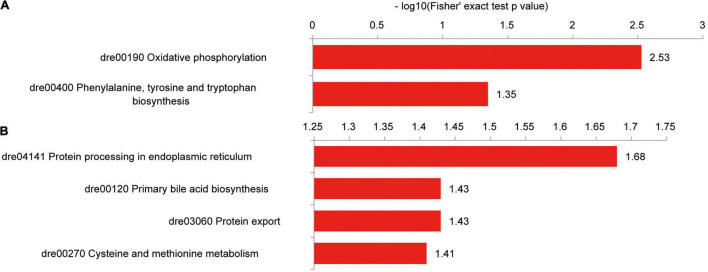
KEGG pathways of differentially succinylated proteins in the liver of zebrafish fed the CK or S0.15 diet for 4 weeks. **(A)** Pathways involved in proteins with increased succinylation. **(B)** Pathways involved in proteins with decreased succinylation.

Hepatic proteins with increased succinylation levels were observed in the oxidative phosphorylation and phenylalanine, tyrosine, and tryptophan biosynthesis pathways, including Atp5a1, Atp5c1, Atp5h, and Got2a proteins (Figure 10A). Proteins with decreased succinylation levels were found within protein processing and transport pathways in the endoplasmic reticulum (prolyl 4-hydroxylase, beta polypeptide, Sec61 translocon subunit alpha 1 (Sec61a1), protein disulfide isomerase family A member 4, Hsp90b1, and Hspa5), the primary bile acid biosynthesis pathway (sterol carrier protein 2a and Hsd17b4) and cysteine and methionine metabolism (betaine-homocysteine methyltransferase, adenosylhomocysteinase, and methionine adenosyltransferase 1A; Figure 10B).

An interaction was observed between Atp5a1, Atp5c1, and Atp5h, which had increased succinylation levels (Supplementary Figure 4A). Proteins with decreased succinylation levels formed three interaction niches involving the ribosome [ribosomal protein L22 (Rpl22), Rplp2l, ribosomal protein S25 (Rps25), Rpl25, Rps11, Rps21, Rps3a, Rpl4, Rpl11, and Sec61a1], glucose metabolic process [glyceraldehyde-3-phosphate dehydrogenase (Gapdh), triosephosphate isomerase 1b, phosphoglucomutase 1, aldehyde dehydrogenase 9 family, member A1a, and enolase 3], and protein processing and transport in the endoplasmic reticulum (Supplementary Figure 4B). Different from the case of the intestine, dietary succinate might lead to decreased levels of protein succinylation in the liver.

### Dietary Succinate Alters the Gut Microbiota Composition of Zebrafish

**FIGURE 11:**
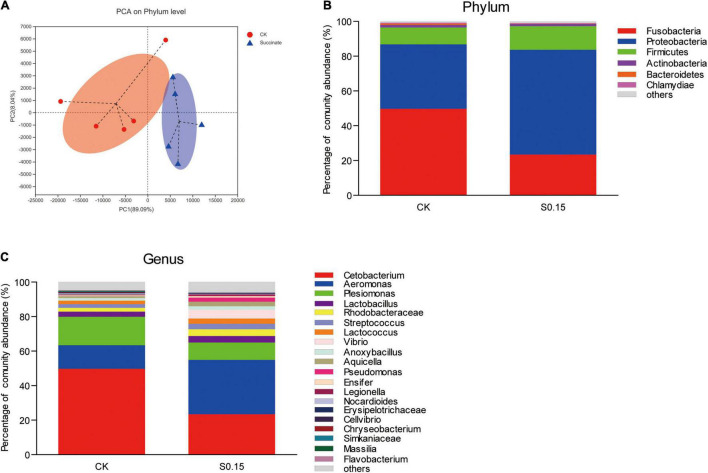
Gut microbial community in zebrafish fed the CK or S0.15 diet for 4 weeks. **(A)** PCA analysis of the gut microbiota. **(B)** Relative abundance at the phylum level of the gut microbial community. **(C)** Relative abundance at the genus level of the gut microbial community. Values are means ± SEMs (*n* = 5 biological replicates). CK, control-check diet; S0.15, 0.15% succinate-supplemented diet.

The α diversity indexes of the gut microbial community, including Shannon, Simpson, and Chao, showed no differences between the S0.15 and the CK group (Table 1). However, PCA analysis indicated a different gut community composition at the OTU level with PC1 variances of 89.09% and PC2 of 8.04% (Figure 11A). At the phylum level, there was a significant increase in the relative abundance of Proteobacteria (60.24 vs. 37.04%, *p* < 0.01) and a reduction in the relative abundance of Fusobacteria (23.38 vs. 49.75%, *p* < 0.01) in the S0.15 group compared with the CK group (Table 2 and Figure 11B). Moreover, the ratio of Fusobacteria to Proteobacteria was significantly decreased in S0.15-fed zebrafish (*p* < 0.05; Table 2). At the genus level, the abundance of *Cetobacterium* (23.38 vs. 49.75%, *p* < 0.01) was significantly reduced in the S0.15 group compared to the CK group (Table 3 and Figure 11C). These results suggest that dietary succinate promotes the growth of Proteobacteria but diminishes Fusobacteria and *Cetobacterium*.

**TABLE 1:** Diversity indexes of the gut bacteria of zebrafish fed with the CK or S0.15 for 4 weeks.

Estimators	Shannon	Simpson	Ace	Chao	Coverage
CK	1.90 ± 0.33	0.32 ± 0.06	250.74 ± 12.23	247.42 ± 15.44	1.00 ± 0.00
S0.15	2.32 ± 0.25	0.23 ± 0.05	256.49 ± 6.65	264.97 ± 10.00	1.00 ± 0.00

*Values are expressed as the mean ± standard error of the mean (SEM), n = 5 biological replicates. CK, control-check diet; S0.15, 0.15% succinate-supplemented diet.*

**TABLE 2:** The predominant gut bacterial phylum in zebrafish fed the CK or S0.15 for 4 weeks based on V3–V4 sequences.

Phylum	CK	S0.15
Fusobacteria	49.75 ± 6.38	23.38 ± 3.38**
Proteobacteria	37.04 ± 5.24	60.24 ± 4.59**
Firmicutes	9.70 ± 2.72	13.59 ± 1.31
Fusobacteria/Proteobacteria	1.55 ± 0.46	0.41 ± 0.07*

Values are expressed as the mean ± standard error of the mean (SEM), n = 5 biological replicates. *p < 0.05, **p < 0.01. CK, control-check diet; S0.15, 0.15% succinate-supplemented diet.

**TABLE 3:** The predominant gut bacterial genus in zebrafish fed the CK or S0.15 for 4 weeks based on V3–V4 sequences.

Genus	CK	S0.15
Cetobacterium	49.75 ± 6.38	23.38 ± 3.38**
Aeromonas	13.59 ± 5.03	31.46 ± 12.06
Plesiomonas	16.50 ± 3.69	10.15 ± 3.57
Lactobacillus	3.04 ± 0.77	3.68 ± 0.48
Rhodobacteraceae	2.14 ± 0.87	3.95 ± 2.49
Streptococcus	2.17 ± 0.58	3.21 ± 0.32
Lactococcus	2.00 ± 0.55	3.01 ± 0.29
Vibrio	0.00 ± 0.00	5.04 ± 5.63
Anoxybacillus	1.53 ± 0.57	2.05 ± 0.45

Values are expressed as the mean ± SEM, n = 5 biological replicates. **p < 0.01. CK, control-check diet; S0.15, 0.15% succinate-supplemented diet.

## Discussion

For the purpose of seeking potential substitutes for antibiotic growth promoters, the application of organic acids in aquaculture has received increasing attention (14). The earliest evaluation of succinate as an aquatic feed additive was conducted in 1988 by Fauconneau, in which rainbow trout fed a 12% succinate-added diet showed reduced growth and feed efficiency despite increased protein efficiency (17). However, recent studies suggest that succinate at an additive amount of 0.5% is an effective feed additive for shrimp, as shown by the promotion of growth, immunity, and digestion (19, 21). The different results in rainbow trout and shrimp might be related to the difference of species and concentrations of succinate used. Therefore, the present study firstly identified the optimal additive amount (0.15%) in zebrafish. The findings in this study are beneficial to improve our understanding of the effects of succinate on growth, feed intake, nutritional metabolism, protein succinylation, and gut microbiota.

In this study, succinate feeding increased the feed intake and growth of zebrafish but had no influence on the feed efficiency and digestive enzyme activities, suggesting that the growth-promoting effects of succinate might be partly related to increased feed intake. Feeding behaviors are regulated through the brain-gut axis, involving multiple hormones and hypothalamic centers (32). Cholecystokinin (CKK) is known to induce satiety and reduce food intake *via* vago-vagal reflexes (32). Notably, succinate has been reported to stimulate the secretion of CKK from duodenal mucosal cells (33). However, in this study, succinate supplementation did promote zebrafish’s appetite, indicating there might be another stimulatory mechanism affecting feed intake. Not only does it act on the gut, but also succinate can pass the blood–brain barrier to boost cerebellar function, thus to improve motor activity, suggesting a direct action on the brain (34). Also, oral administration of succinate can increase the amount of food intake in mice (35). A previous study using another organic acid, acetate, has shown a parasympathetic nerve-dependent stimulation mechanism, whereby both acetate feeding and intracerebroventricular injection can promote fish feed intake and upregulate orexigenic genes in the brain (15). Therefore, we speculate that the appetite-promoting effects of succinate might be related to a gut-brain mode in zebrafish.

With increased feed intake, zebrafish gained more protein, lipid, or other nutrients. The efficiency of these nutrients is affected by anabolic and catabolic processes. In this study, zebrafish fed the S0.15 diet deposited more protein and lipids, suggesting elevated anabolism over catabolism. The TCA cycle is the center for glucose, lipid, and protein metabolism. Pyruvate, fatty acids, and amino acids fuel the mitochondrial TCA cycle by providing acetyl-CoA or other anaplerotic inputs (36). Intermediates of the TCA cycle, such as malate, oxaloacetate, and citrate, can be exported from mitochondria into cytosol for the synthesis of glucose, fatty acids, or amino acids (37–40). Succinate is an important intermediate of the TCA cycle (41). Thus, the surplus or absence of succinate will alter the direction or function of the TCA cycle, thus influencing nutritional metabolism.

To further define the effects of succinate on nutritional metabolism, we measured the levels of succinate, pyruvate, and acetyl-CoA in the intestine, liver, and muscle. In this study, succinate and acetyl-CoA levels in the intestine increased when succinate was incorporated into the diet. Elevated succinate might either lead to the feedback inhibition of α-ketoglutarate dehydrogenase and the accumulation of α-KG, isocitrate, and citrate, or stimulate the TCA cycle by increasing the synthesis of fumarate, malate, and oxaloacetate (42, 43). Citrate acts as a direct precursor of cytosolic acetyl-CoA that is used for *de novo* lipid synthesis (39, 40). In accordance to the increased level of acety-CoA, lipid synthesis in the intestine was increased by dietary succinate, as shown by the upregulation of genes related to fatty acid and TG synthesis.

Malate is the precursor of pyruvate and can be used for gluconeogenesis (44, 45). In contrast to the increased intestinal pyruvate level, gluconeogenesis in the intestine was inhibited by dietary succinate as evidenced by the downregulation of gluconeogenesis-related genes, suggesting the existence of another substrate-independent modulation toward intestinal gluconeogenesis, such as protein posttranslational modification. It has been suggested that histone acetylation and memethylation can positively regulate gluconeogenesis by promoting the expression of glucose-6-phosphatase and PCK (46, 47), while the acetylation and phosphorylation of forkhead box class O1 (FoxO1) negatively regulate the transcriptional expression of gluconeogenic genes (48, 49). Similar to acetate, succinate entering cells can be converted to succinyl-CoA and enhance lysine succinylation (50–52). In addition, histone succinylation tends to stimulate gene transcription (53). However, no transcriptional factors like FoxO1 were identified with succinylation in this study. Therefore, transcriptional suppression of gluconeogenic genes in the intestine might be related to other types of protein modification, which requires further study.

Importantly, intestinal gluconeogenesis plays a central role in controlling glucose homeostasis (54). Mammalian studies have suggested that intestinal gluconeogenesis stimulated by protein-enriched diets can lead to increased glucose release into the portal vein (55). Glucose sensors detect portal glucose concentrations and initiate a neuroendocrine-mediated suppression to food intake and hepatic gluconeogenesis (54). Impaired intestinal gluconeogenesis induces fasting hyperglycemia and hyperinsulinemia, which can be rescued by portal glucose infusion or inhibition of α-2 adrenergic receptors, confirming the gut-brain neural circuit controlled by intestinal gluconeogenesis (56). Therefore, the reduced intestinal gluconeogenesis observed in zebrafish fed S0.15 diet might be closely linked to increased hepatic gluconeogenesis through a gut-brain neuroendocrine axis. Moreover, it is remarkable that intestinal gluconeogenesis correlates negatively with the food intake of mice (54, 55). Mice with impaired intestinal gluconeogenesis are less sensitive to satiety induced by a protein-enriched diet and ingest more food (57). Thus, the increased feed intake of fish fed the S0.15 diet might be partly associated with the intestinal gluconegenesis-controlled gut-brain neural circuit.

In this study, despite the promotion of hepatic gluconeogenesis, plasma glucose decreased and glucose tolerance increased in fish fed a succinate-supplemented diet, suggesting improved glucose homeostasis. Hepatic gluconeogenesis primarily accounts for the elevation of blood glucose (58, 59). In addition to the postprandial increase in blood glucose, hepatic glycogenesis can also be promoted by increased hepatic gluconeogenesis or the input of glycogenic substrates (60). GYS is the rate-limiting enzyme of glycogen synthesis. Its activation by glucose-6-phosphate (G6P) in the liver has been reported to improve glucose homeostasis (61, 62). The muscle is considered to be another primary tissue for glycogen synthesis, which takes up glucose *via* three steps: glucose delivery from the circulation to the muscle, glucose transport across the muscle membrane, and the production of G6P (63). Similar to the liver, elevated intracellular contents of G6P can activate glycogen synthase in the muscle (64). Many fish studies have suggested that fish are capable to store excess blood glucose in the form of hepatic and muscle glycogen synthesis (65–67). Therefore, we speculate that increased glycogen synthesis in the liver and muscle might partly contribute to improved glucose homeostasis.

In the liver, the level of succinate was decreased with dietary succinate supplementation. The reason for this reduction is unknown. However, succinate is the precursor for propionate production of the gut microbiota (68). In addition, an *in vitro* study has suggested that succinate can be efficiently metabolized to CO_2_, amino acids, pyruvate, and lactate by Caco2 cells (69). Thus, we cannot exclude that part of succinate might be utilized by intestinal epithelial cells or the gut microbiota before it can enter the liver. Furthermore, succinate could flow into the gluconeogenesis pathway *via* the malate-aspartate shuttle in the liver (44, 45). Combined with the upregulation of hepatic gluconeogensis-related genes, we can hypothesize that the reduction in hepatic succinate might be partly due to the improvement of hepatic gluconeogenetic flux.

Despite the reduction of succinate in the liver, increased nutrient intake resulting from the appetite-promoting effects of succinate might continuously contribute to fuel the TCA cycle, likely increasing the synthesis of α-KG, isocitrate, and citrate. Similarly, increased citrate might be transported to cytosol and participate in hepatic lipogenesis. The level of hepatic acetyl-CoA was increased with dietary succinate supplementation, consistent with increased hepatic lipid synthesis. Taken together, the accumulation of whole body fat in fish fed a succinate-supplemented diet results mainly from intestinal and hepatic lipid accumulation.

Additionally, succinate feeding can also increase whole body protein deposition in zebrafish by reducing protein catabolism, as shown by the downregulation of protein degradation-related genes in the intestine, liver, and muscle, suggesting a protein-sparing effect of dietary succinate. This effect has also been observed in rainbow trout, in which 12% succinate supplementation improved protein efficiency (17). For achieving the protein-sparing effect, it is important to increase energy supply from non-protein catabolism, such as lipids and carbohydrates (70, 71). For example, diacylglycerol oil exerts a protein-sparing effect by promoting lipid catabolism in Nile tilapia (72). In this study, dietary succinate promoted muscle glycolysis. Thus, we can speculate that one of the reasons for the protein-sparing effect of succinate is the improved glucose utilization efficiency in muscle, consistent with improved glucose homeostasis. Although lipid and glucose catabolism in the intestine and liver of fish fed a succinate-supplemented diet showed no improvement, the FADH_2_-producing step of succinate oxidation that couples with electron transport chain by providing protons for ATP generation (73) might partly account for the protein-sparing effect of succinate in the intestine and liver.

Consistent with increased feed intake, increased body weight gain, and whole body fat and protein deposition, the whole body energy gain was promoted by succinate feeding. In addition, fish fed the S0.15 diet showed a reduction in standard metabolic energy. Standard metabolic energy refers to energy expenditure for organ maintenance, nutrient assimilation, and adaptation to changes in resource availability (74). In free-living pike (*Esox lucius*), standard metabolic energy comprises up to 90% of the energy budget (75). Moreover, zebrafish with lower standard metabolic rates tend to have higher growth rates (25). The increased energy gain in fish fed a succinate-supplemented diet might be partly associated with reduced energy expenditure in the form of standard metabolic energy.

Lysine succinylation is a newly identified post-translational modification that is conserved from prokaryotes to eukaryotes (52). Global succinylation has been described in zebrafish and suggests a major regulatory role in several metabolic processes such as carbon metabolism and the TCA cycle (76). Generally, proteins in the TCA cycle or closely interacting with the TCA cycle are more readily accessible to succinyl-CoA and to be succinylated (76, 77). Upon succinate feeding, increased protein succinylation was observed in proteins in Cs, Mdh2, Idh3b, and Pck2, while reduced protein succinylation was observed in Hadhaa, Hadhab, and Gapdh.

Existing studies have suggested that succinylation tends to compromise protein function. De-succinylation of IDH2, pyruvate kinase 2, 3-hydroxy-3-methylglutaryl-CoA synthase 2, and CS by sirtuin 5 recovers their enzymatic activities (78–81). Thus, the intestinal TCA cycle might be inhibited partly by the succinylation of enzymes (Cs, Mdh2, and Idh3). However, Hadhaa and Hadhab are not the same case. Acetylation of HADHAA and HADHAB of mice activates their enzymatic activities (82). Thus, we speculate that reduced succinylation of Hadhab and Hadhab of zebrafish might lead to their inactivation and the inhibition of hepatic fatty acid degradation. In addition, a previous study has suggested that de-malonylation can activate GAPDH and maintain glycolysis (83). Here, we speculate that reduced succinylation of Gapdh might promote hepatic glycolysis and glucose utilization. As the effects of acylation on protein function are related to protein structures, sites, charges, and polarity, further studies are needed to investigate the relationships between succinylation and these target proteins.

However, the findings related to the modulation of metabolism in this study are opposed to previous studies on mice. It was reported that succinate protects against diet-induced obesity by an uncoupling protein (UCP1)-dependent thermogenesis of brown and beige adipose tissue and promotes energy expenditure in mice (43, 84). UCP1 accounts for the direct conversion of nutrient energy into heat (85). However, zebrafish have no brown adipocyte tissues and are unable to produce adipocyte browning under cold stress, heat stress, or chemical stimulation (86). Considering the physiological differences between mammals and fish, the results of mammalian studies cannot be directly transited to zebrafish.

In fish, the gut microbial community is highly sensitive to dietary factors (87) and could influence food ingestion and absorption, metabolism, immune response, energy homeostasis, and the health of fish (88). In this study, dietary succinate increased the enrichment of Proteobacteria, but diminished that of Fusobacteria and *Cetobacterium*. It has been suggested that Proteobacteria is a microbial signature of dysbiosis in the gut microbiota (89) and dominates the microbiota of unhealthy fish (90). Moreover, the abundance of *Cetobacterium* is much higher in healthy fish than in diseased fish (91). Although no apparent effects on fish health were observed, alterations in the abundance of Proteobacteria and *Cetobacterium* suggest a potential gut microbiota dysbiosis induced by dietary succinate. A healthy intestinal mucosa can be measured by “physiologic hypoxia,” which is characterized by a steep oxygen gradient from the lumen to the serosa, along the length of the intestine (92). The shift in luminal oxygen concentration is regarded as one of the reasons accounting for the reduction of obligate anaerobes, such as *Cetobacterium*, and the expansion of oxygen-tolerant species including members of Proteobacteria (27, 89). Therefore, we can hypothesize that dietary succinate might impair “physiologic hypoxia” in zebrafish.

Inflammation is one of the factors resulting in increased oxygen gradient in the intestinal lumen as a result of increased blood flow and vascular permeability (93). Succinate accumulation has been thought to be a metabolic signal of inflammation (5). Through the inhibition of prolyl hydroxlases, intracellular succinate stabilizes HIF-1α and induces the expression of target genes, including the pro-inflammatory cytokine IL-1β (4). In addition, extracellular succinate can initiate inflammation signaling through the activation of SUR1 (5). LPS-induced succinate accumulation has been suggested to contribute to the production of pro-inflammation cytokines in macrophages (94, 95). Further studies are needed to define whether succinate feeding could induce intestinal inflammation in zebrafish.

## Conclusion

Dietary succinate promotes fish growth, whole body energy deposition, fat accumulation, improves glucose homeostasis, and reduces protein degradation. Intestinal and hepatic fat accumulation contribute to the whole body fat deposition. This is related to increased precursor of lipogenesis, the upregulation of lipid synthesis-related genes, and the downregulation of lipid-degradation-related genes. Improved glucose homeostasis is related to decreased intestinal gluconeogenesis, increased hepatic glycogen, muscle glycogen synthesis, and muscle glycolysis. Dietary succinate promotes whole body protein deposition by downregulating genes related to intestinal, hepatic, and muscular protein degradation. Notably, dietary succinate promotes the growth of Proteobacteria, but diminishes that of Fusobacteria and *Cetobacterium* in zebrafish. This study suggests that dietary succinate could promote growth and feed intake, promote lipid anabolism, improve glucose homeostasis, and spare protein. Alterations in the levels of metabolic intermediates, transcriptional regulation, and protein succinylation appear to be closely associated with the effects of succinate on nutritional metabolism. However, hepatic fat accumulation and gut microbiota dysbiosis induced by dietary succinate suggest potential risks of succinate application as a feed additive for zebrafish.

## Data Availability Statement

The datasets presented in this study can be found in online repositories. The names of the repository/repositories and accession number(s) can be found below: https://www.ncbi.nlm.nih.gov/, PRJNA791519.

## Ethics Statement

The animal study was reviewed and approved by Institute of Feed Research of Chinese Academy of Agricultural Sciences Animal Care Committee.

## Author Contributions

ZGZ and ZZ designed the research and gave conceptual advice for the research. QD and CL performed the experiments and acquired the data. QH and QZ participated in zebrafish husbandry and sampling. CR and YY co-analyzed and discussed the results. QD wrote this manuscript. ER and RO reviewed and helped to revise the manuscript. All authors read and approved the final manuscript.

## Conflict of Interest

The authors declare that the research was conducted in the absence of any commercial or financial relationships that could be construed as a potential conflict of interest.

## Publisher’s Note

All claims expressed in this article are solely those of the authors and do not necessarily represent those of their affiliated organizations, or those of the publisher, the editors and the reviewers. Any product that may be evaluated in this article, or claim that may be made by its manufacturer, is not guaranteed or endorsed by the publisher.
