# Supplementary material for: Dietary Succinate Impacts the Nutritional Metabolism, Protein Succinylation and Gut Microbiota of Zebrafish
Source: Front Nutr. 2022 May 23;9:894278. doi: 10.3389/fnut.2022.894278 (PMC9171437; doi:10.3389/fnut.2022.894278)
Supplement: Supplementary file 1 [file Table_1.pdf]

Supplemental Tables

Supplemental Table 1. Ingredients of experimental diets for one-month-old zebrafish (g/kg).

	One-month-old zebrafish				
Ingredient, g/kg dry diet	CK	S0.05	S0.1	S0.15	S0.2
Casein	400	400	400	400	400
Gelatin	100	100	100	100	100
Wheat flour	280	280	280	280	280
Soybean oil	69.9	69.9	69.9	69.9	69.9
Lysine	3.30	3.30	3.30	3.30	3.30
Ascorbyl phosphater	1.00	1.00	1.00	1.00	1.00
Vitamin mix[1]	2.00	2.00	2.00	2.00	2.00
Mineral mix[2]	2.00	2.00	2.00	2.00	2.00
Calcium Dihydrogen Phosphate	20.0	20.0	20.0	20.0	20.0
Choline chloride	2.00	2.00	2.00	2.00	2.00
Sodium alginate	20.0	20.0	20.0	20.0	20.0
Microcrystalline cellulose	40.0	40.0	40.0	40.0	40.0
Zeolite powder	69.7	69.2	68.7	68.2	67.7
Disodium succinate	-	0.50	1.00	1.50	2.00
Total	1000	1000	1000	1000	1000

[1]Vitamin premix (g/kg): thiamine, 0.438; riboflavin, 0.632; pyridoxine·HCl, 0.908; *d*-pantothenic acid, 1.724; nicotinic acid, 4.583; biotin, 0.211; folic acid, 0.549; vitamin B-12, 0.001; inositol, 21.053; menadione sodium bisulfite, 0.889; retinyl acetate, 0.677; cholecalciferol, 0.116; *dl*-α-tocopherol-acetate, 12.632;

[2]Mineral premix (g/kg): $CoCl_2 \cdot 6H_2O$, 0.074; $CuSO_4 \cdot 5H_2O$, 2.5; $FeSO_4 \cdot 7H_2O$, 73.2; NaCl, 40.0; $MgSO_4 \cdot 7H_2O$, 284.0; $MnSO_4 \cdot H_2O$, 6.50; KI, 0.68; Na_2SeO_3, 0.10; $ZnSO_4 \cdot 7H_2O$, 131.93; Cellulose, 501.09;

[3]Disodium succinate: Sigma, USA

CK, control-check diet; S0.05, S0.1, S0.15 and S0.2, 0.05%, 0.1%, 0.15% and 0.2% succinate-supplemented diets.

Supplemental Table 2. Ingredients of experimental diets for zebrafish larvae (g/kg).

Ingredient, g/kg dry diet	Zebrafish larvae	
	CK	S0.15
Casein	460	460
Geltin	110	110
Wheat flour	240	240
Soybean oil	35.0	35.0
Cod liver oil	35.0	35.0
Soybean lecithin	20.0	20.0
Lysine	3.70	3.70
Ascorbyl phosphater	1.00	1.00
Vitamin premix[1]	2.00	2.00
Mineral premix[2]	2.00	2.00
Calcium Dihydrogen Phosphate	20.0	20.0
Choline chloride	2.00	2.00
Sodium alginate	20.0	20.0
Microcrystalline Cellulose	20.0	20.0
Zeolite powder	29.3	27.8
Disodium succinate[3]	0.00	1.50
Total	1000	1000

[1]Vitamin premix (g/kg): thiamine, 0.438; riboflavin, 0.632; pyridoxine·HCl, 0.908; *d*-pantothenic acid, 1.724; nicotinic acid, 4.583; biotin, 0.211; folic acid, 0.549; vitamin B-12, 0.001; inositol, 21.053; menadione sodium bisulfite, 0.889; retinyl acetate, 0.677; cholecalciferol, 0.116; *dl*-α-tocopherol-acetate, 12.632;

[2]Mineral premix (g/kg): $CoCl_2 \cdot 6H_2O$, 0.074; $CuSO_4 \cdot 5H_2O$, 2.5; $FeSO_4 \cdot 7H_2O$, 73.2; NaCl, 40.0; $MgSO_4 \cdot 7H_2O$, 284.0; $MnSO_4 \cdot H_2O$, 6.50; KI, 0.68; Na_2SeO_3, 0.10; $ZnSO_4 \cdot 7H_2O$, 131.93; Cellulose, 501.09;

[3]Disodium succinate: Sigma, USA

CK, control-check diet; S0.15, 0.15% succinate-supplemented diet.

Supplemental Table 3. Quantitative PCR primers.

Gene	Forward primer（5'-3'）	Reverse primer (5'-3'）
rps11	acagaaatgccccttcactg	gcctcttctcaaaacggttg
g6pc1a	tggcagtgataggagattggctt	agtaggacgtctcatggacccac
pck1	atcgcatcacgcatcgctaaa	ccgctgcgaaatacttcttctgt
pfk	gtaacacgcatgggcatttttg	tcgccagtttgatgtgatctcct
pk	atcactgcccgcaacacca	tcattcctgctttcaccatctcc
gys2	ttgaagatctcctgctctttgagg	cattcgtccacagtgatctttgct
acc1	gcgtggccgaacaatggcag	gcaggtccagcttccctgcg
fas	ggagcaggctgcctctgtgc	ttgcggcctgtcccactcct
dgat2	acgcataacctgcttccc	tcctgtggcttctgtccc
cpt1a	catccttaggcctgctcttcaaa	accatgacaccccccaactaacat
acox3	tggaaggacatgatgcgcttt	aggctgccgggcaaaaa
ehhadh	gaatacttgtgaggtggctctgga	aggacacggtgtggtcagcat
mtor	tgggagcagacaggaatgaagg	tgcacctgctggaaaaagaatg
pept1	tggtgaatgagttctgtgagcga	acaggtcatcatcccaaccaatg
asns	ttcagaatgctgactgacgatgg	tggaaaagcagtgatctttgcag
gdh1a	aggacattgtgcattcgggatt	cctcagatccagcccaaggttat
apn	ggtggcttttaccggagtgaata	caaggaaatgcttttctggcatc
