# Supplementary material for: Dietary Succinate Impacts the Nutritional Metabolism, Protein Succinylation and Gut Microbiota of Zebrafish
Source: Front Nutr. 2022 May 23;9:894278. doi: 10.3389/fnut.2022.894278 (PMC9171437; doi:10.3389/fnut.2022.894278)
Supplement: Supplementary file 2 [file Image_1.pdf]

Supplemental Figures

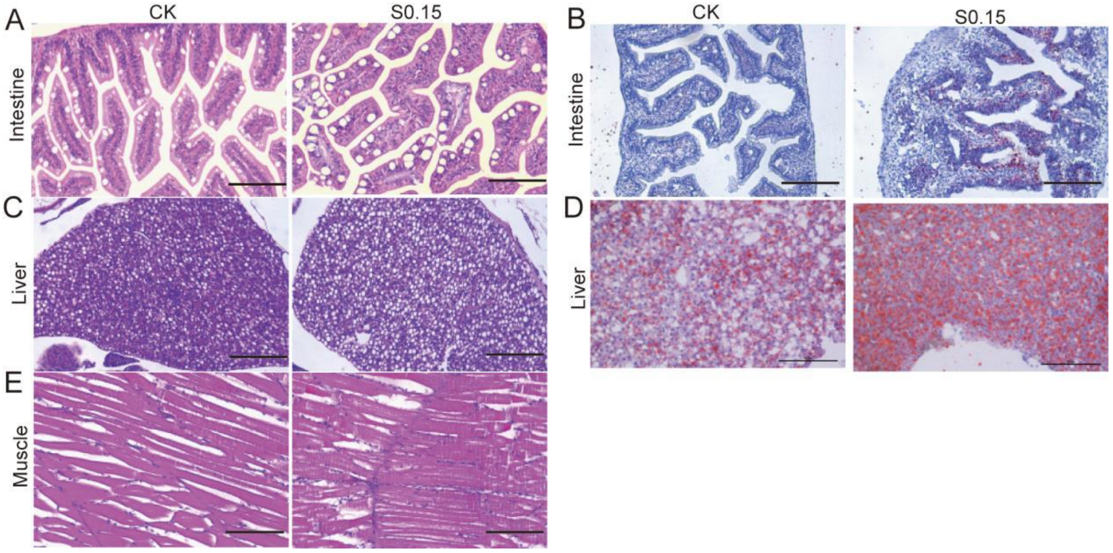

Supplemental Figure 1. Effects of dietary succinate on histological changes and fat accumulation in zebrafish fed CK or S0.15 for 4 weeks. (A) H&E and (B) oil red O staining section of the intestine. (C) H&E and (D) oil red O staining section of the liver. (E) H&E staining section of the muscle. CK, control-check diet; S0.15, 0.15% succinate-supplemented diet. Scale bar, 100 μm.

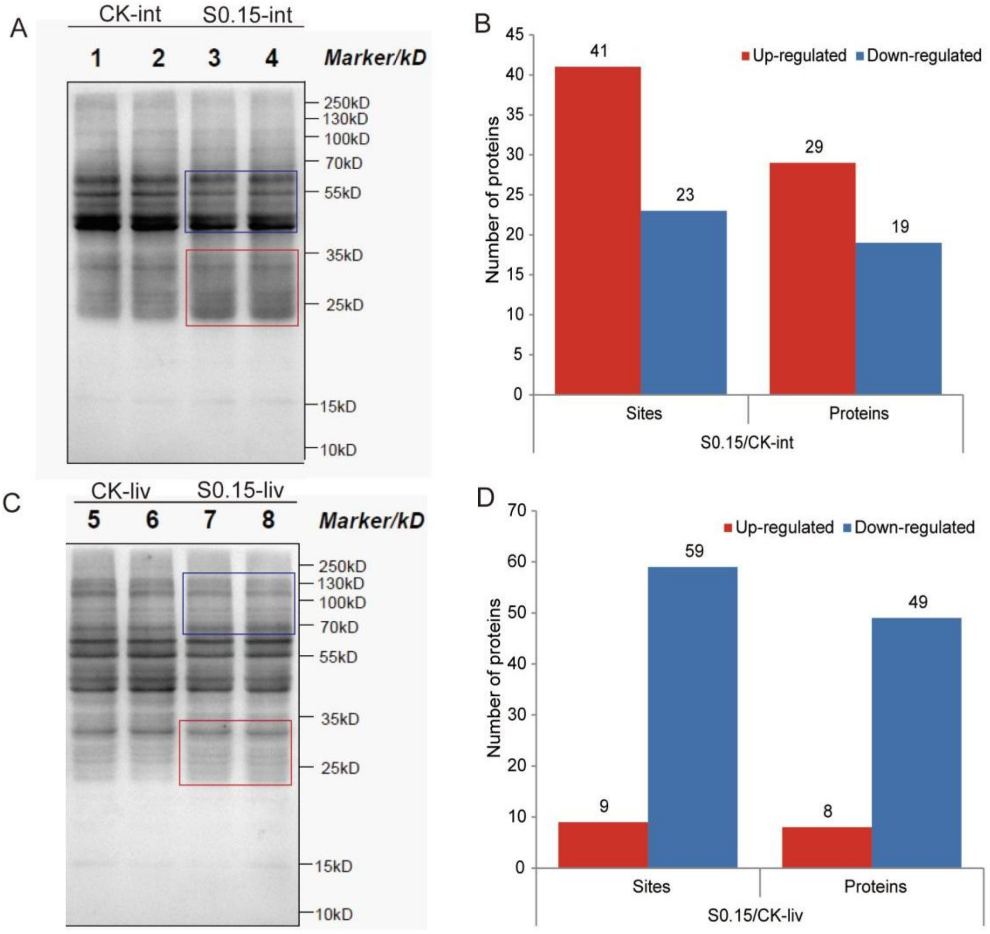

Supplemental Figure 2. Effects of dietary succinate on protein succinylation profiling in zebrafish fed CK or S0.15 diet for 4 weeks. (A) Representative western blot of intestinal protein succinylation confirmed by using an anti-succinyllysine antibody. (B) Summary of all differentially modified proteins and sites in intestine. (C) Representative western blot of hepatic protein succinylation confirmed using an anti-succinyllysine antibody. (D) Summary of all differentially modified proteins and sites in liver. Values are means ± SEMs (n = 3 biological replicates). CK, control-check diet; S0.15, 0.15% succinate-supplemented diet. Int, intestine; Liv, liver.

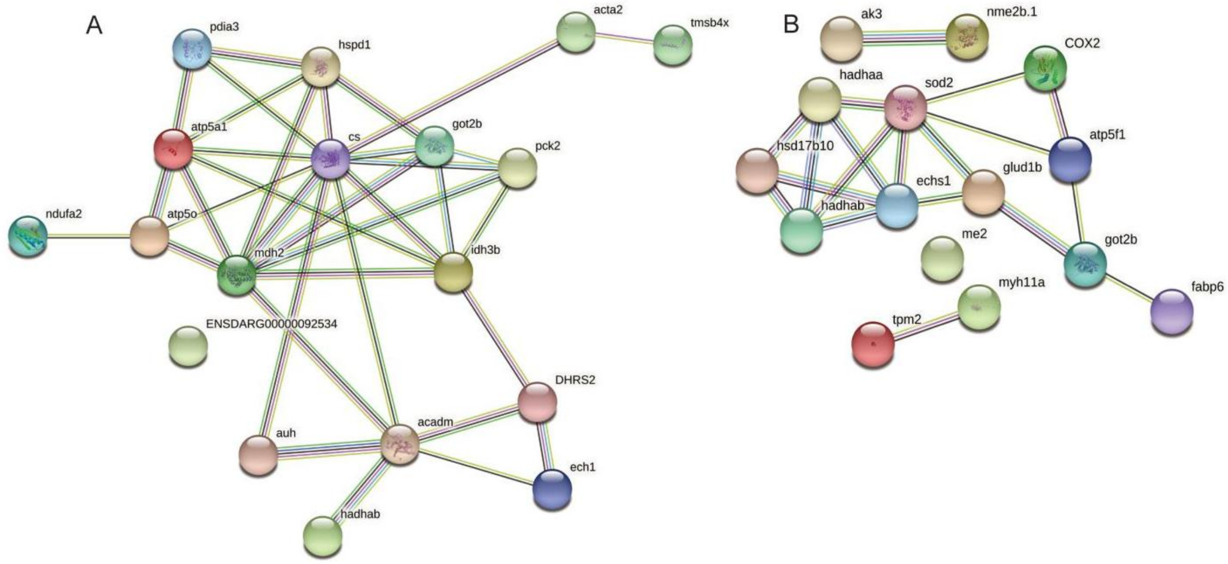

Supplemental Figure 3. STRING analysis of differentially succinylated proteins in the intestine of zebrafish fed CK or S0.15 diet for 4 weeks. (A) Protein interaction network involved with proteins with elevated succinylation in intestine. (B) Protein interaction network involved with proteins with reduced succinylation in intestine. CK, control-check diet; S0.15, 0.15% succinate-supplemented diet.

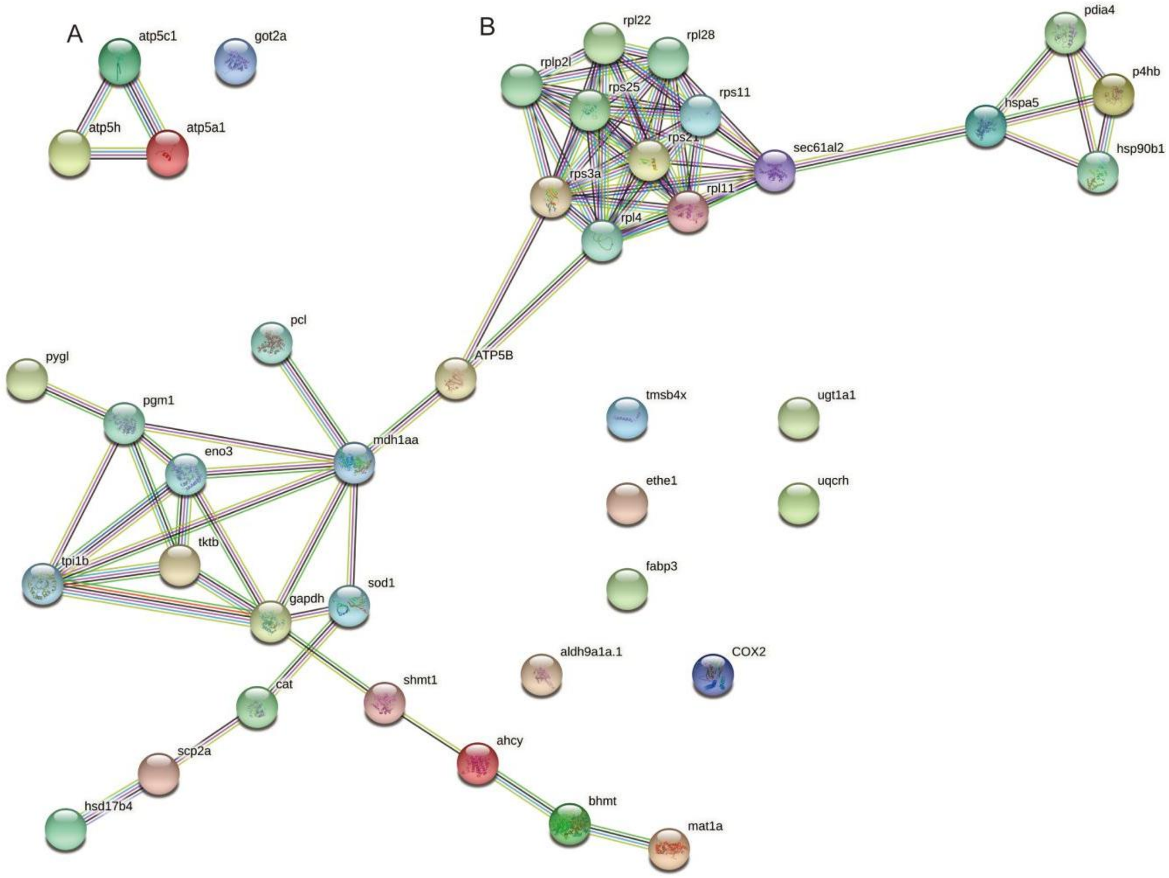

Supplemental Figure 4. STRING analysis of differentially succinylated proteins in the liver of zebrafish fed CK or S0.15 diet for 4 weeks. (A) Protein interaction network involved with proteins with elevated succinylation in liver. (B) Protein interaction network involved with proteins with reduced succinylation in liver. CK, control-check diet; S0.15, 0.15% succinate-supplemented diet.
